# Supplementary figures and images for: The co-expression networks of differentially expressed RBPs with TFs and LncRNAs related to clinical TNM stages of cancers
Source: PeerJ. 2019 Sep 17;7:e7696. doi: 10.7717/peerj.7696 (PMC6753928; doi:10.7717/peerj.7696)

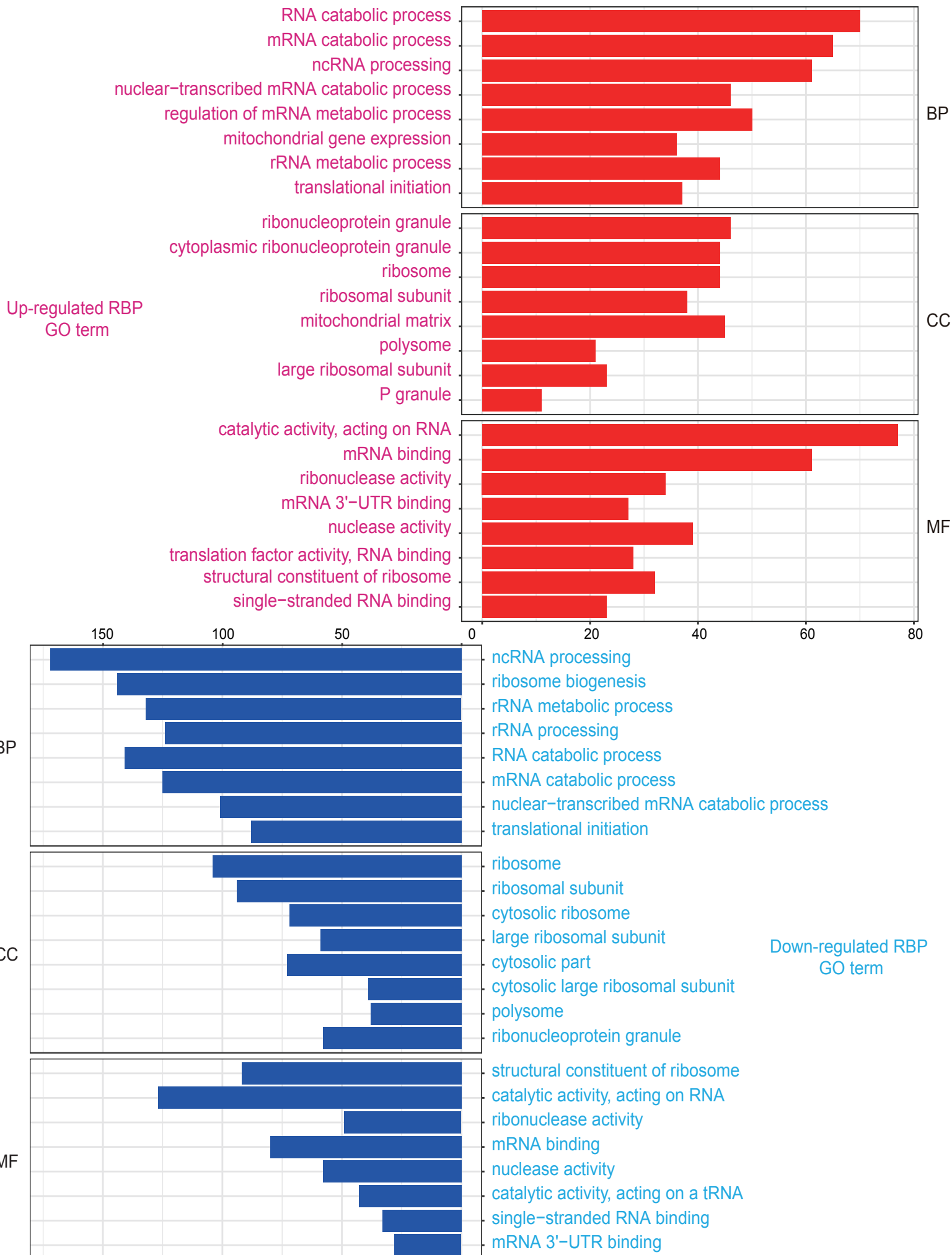

Supplement: Figure S1 [file peerj-07-7696-s001.pdf]

(A) BLCA.Module-trait relationships

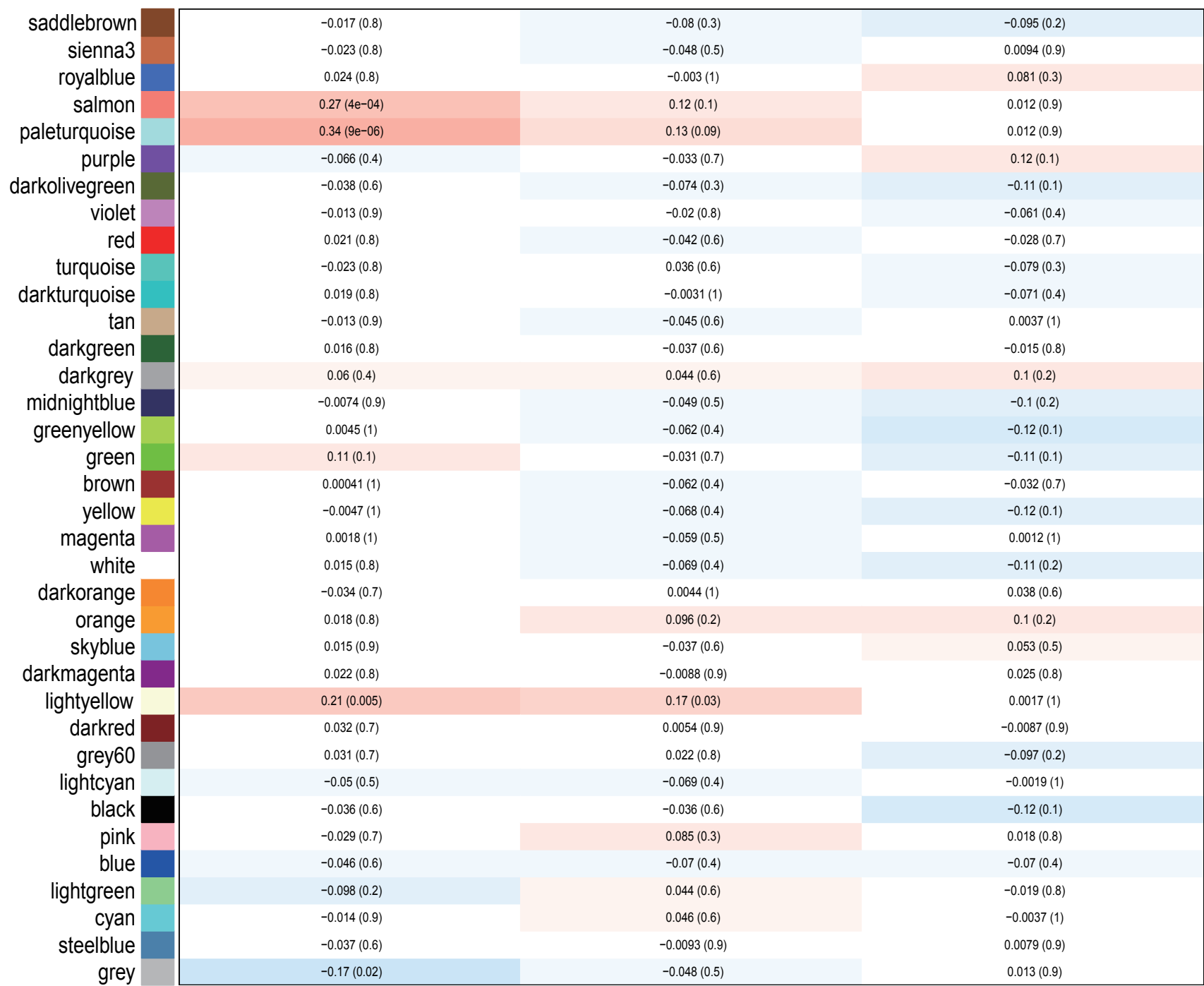

(B) BRCA.Module-trait relationships

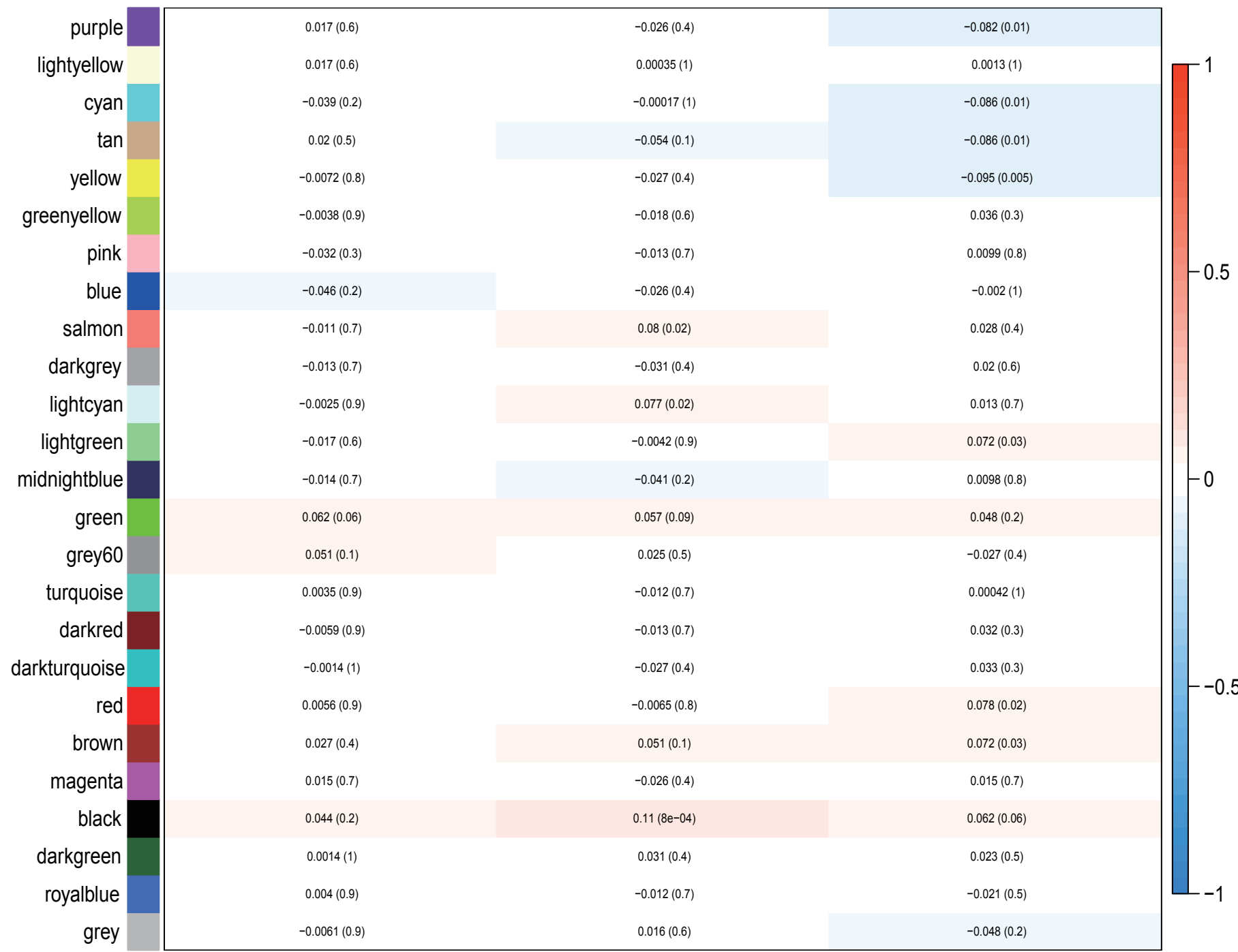

Supplement: Figure S2 [file peerj-07-7696-s002.pdf]

(C) CESC.Module-trait relationships

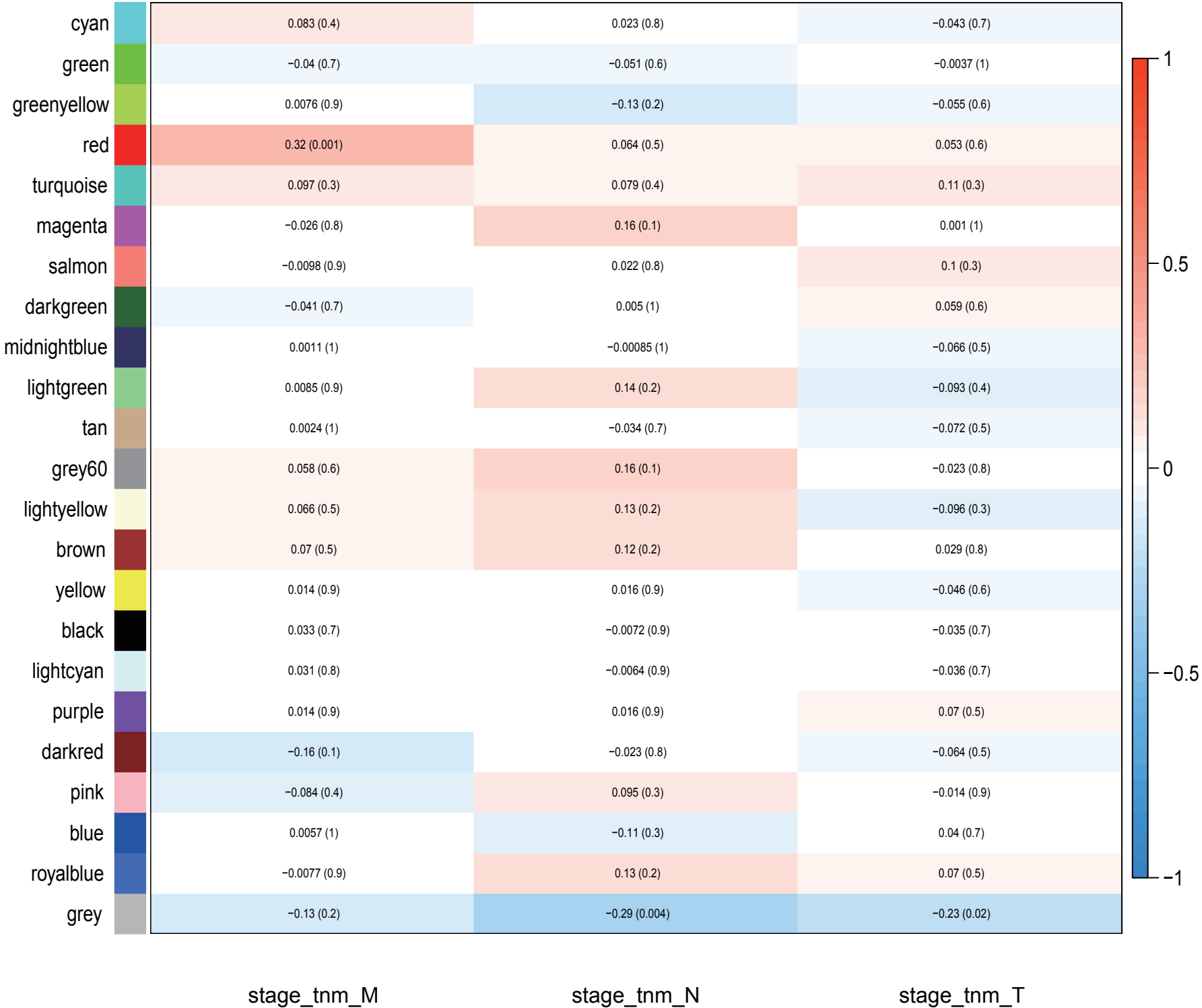

(D) COAD.Module-trait relationships

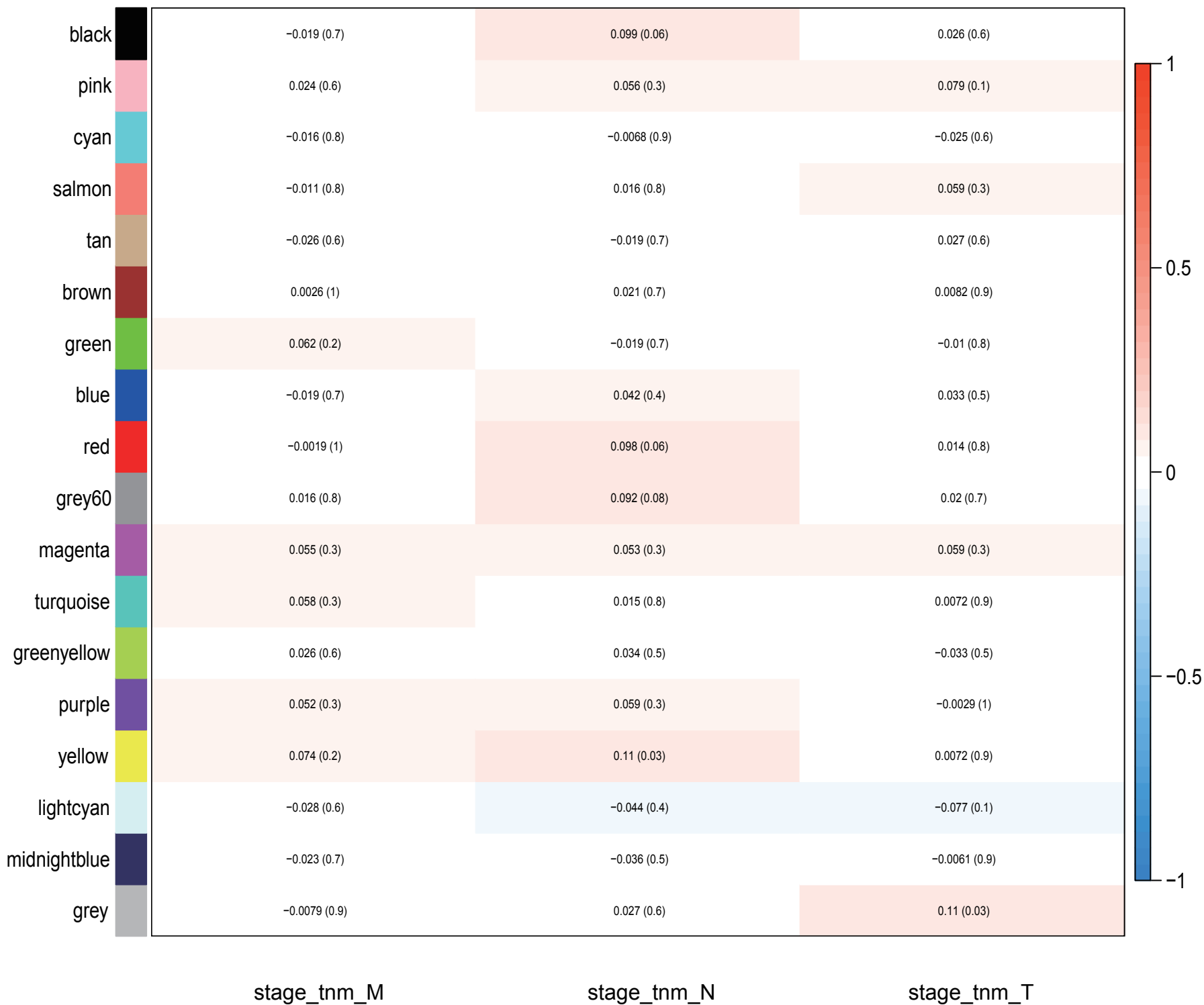

Supplement: Figure S3 [file peerj-07-7696-s003.pdf]

(E) KIRC.Module-trait relationships

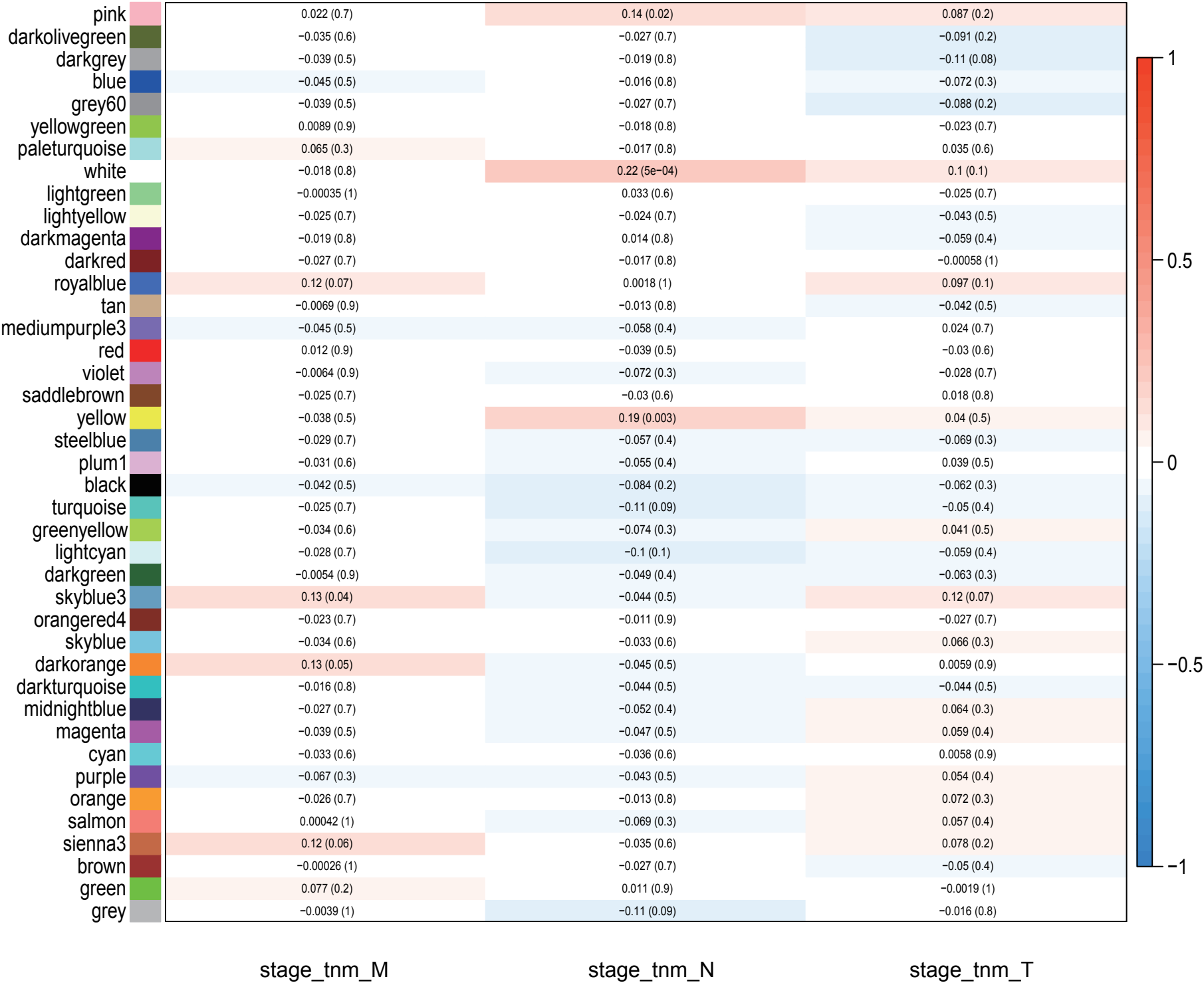

(F) KIRP.Module-trait relationships

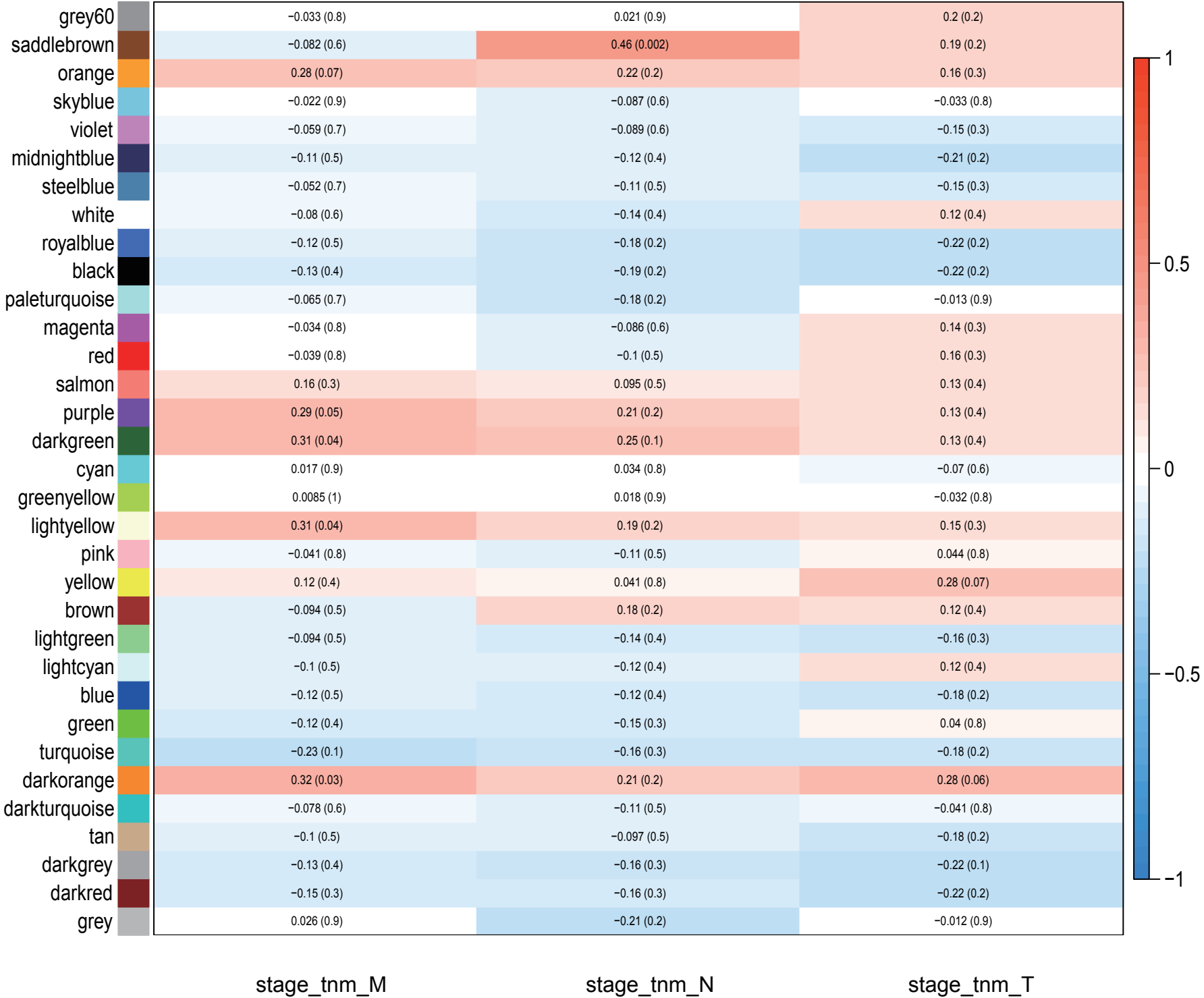

Supplement: Figure S4 [file peerj-07-7696-s004.pdf]

(G) LIHC.Module-trait relationships

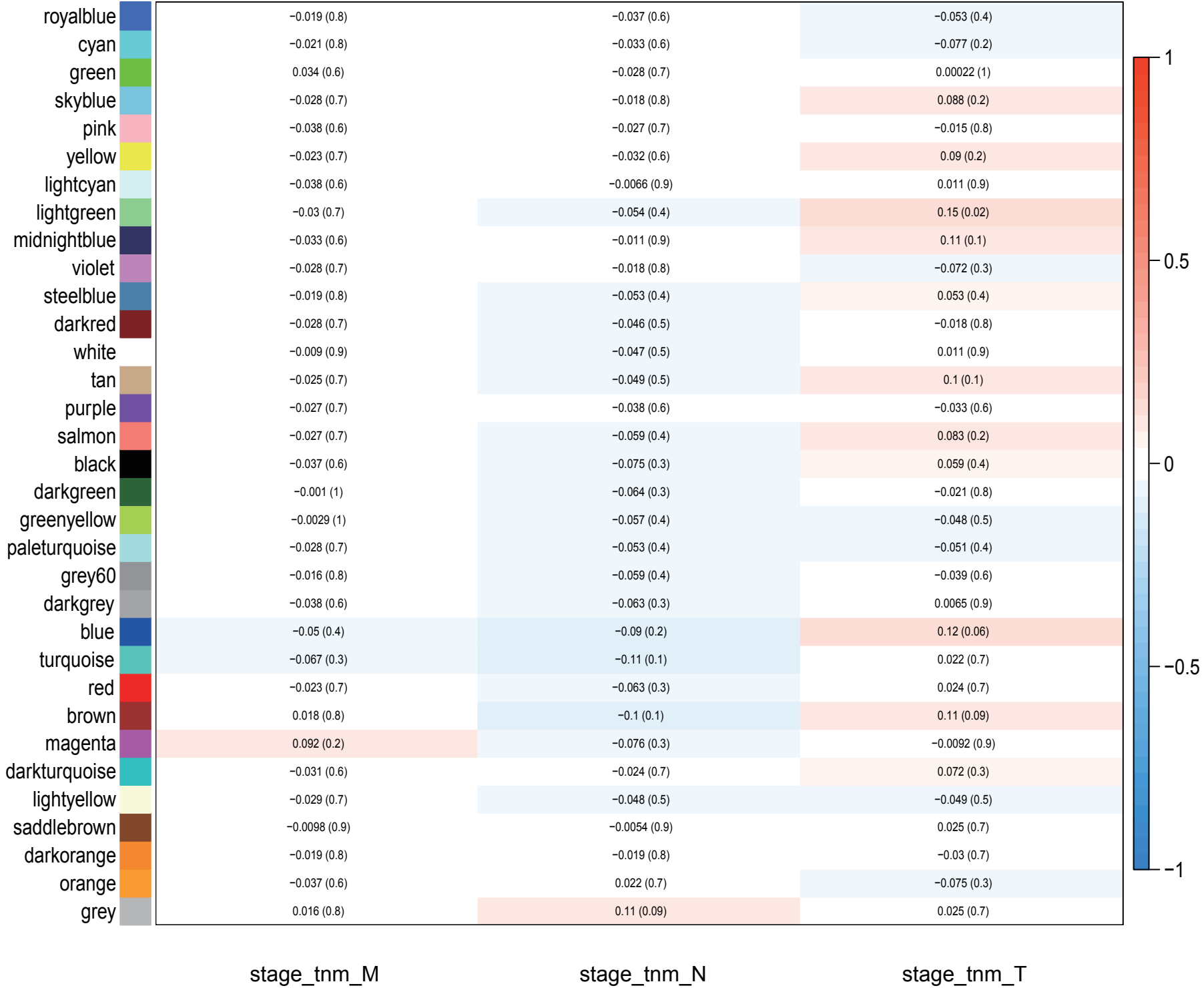

(H) LUAD.Module-trait relationships

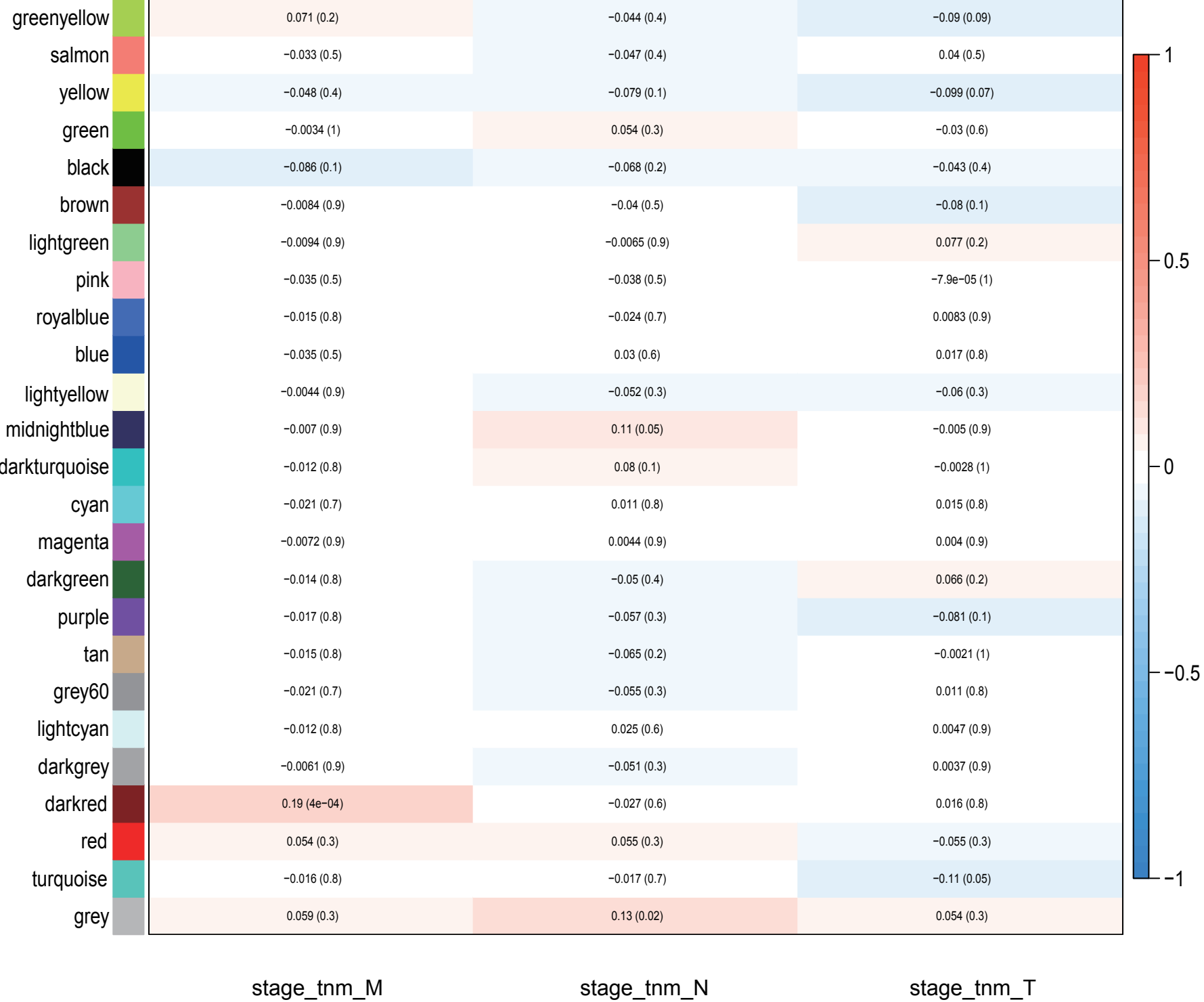

Supplement: Figure S5 [file peerj-07-7696-s005.pdf]

(I) LUSC.Module-trait relationships

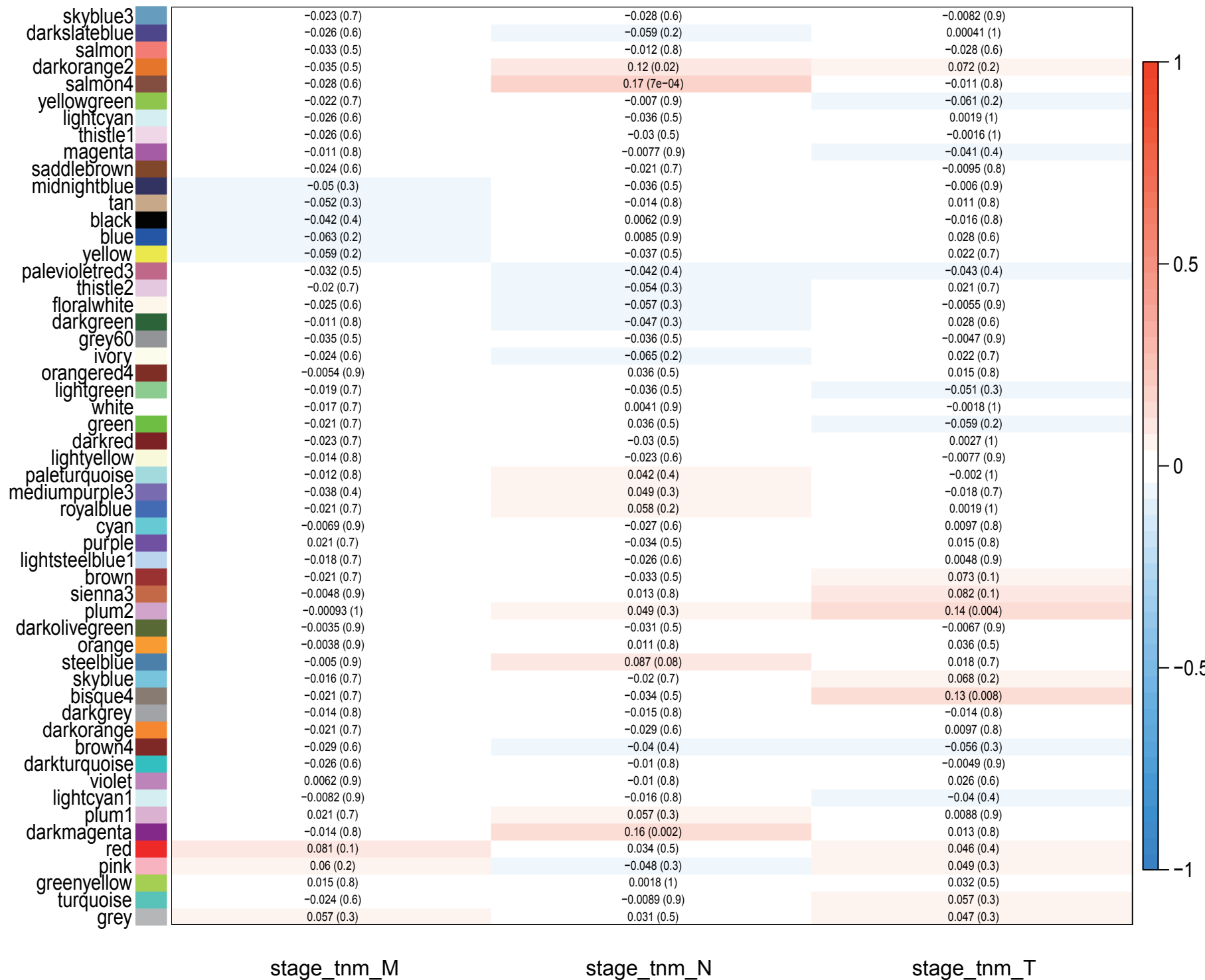

(J) READ.Module-trait relationships

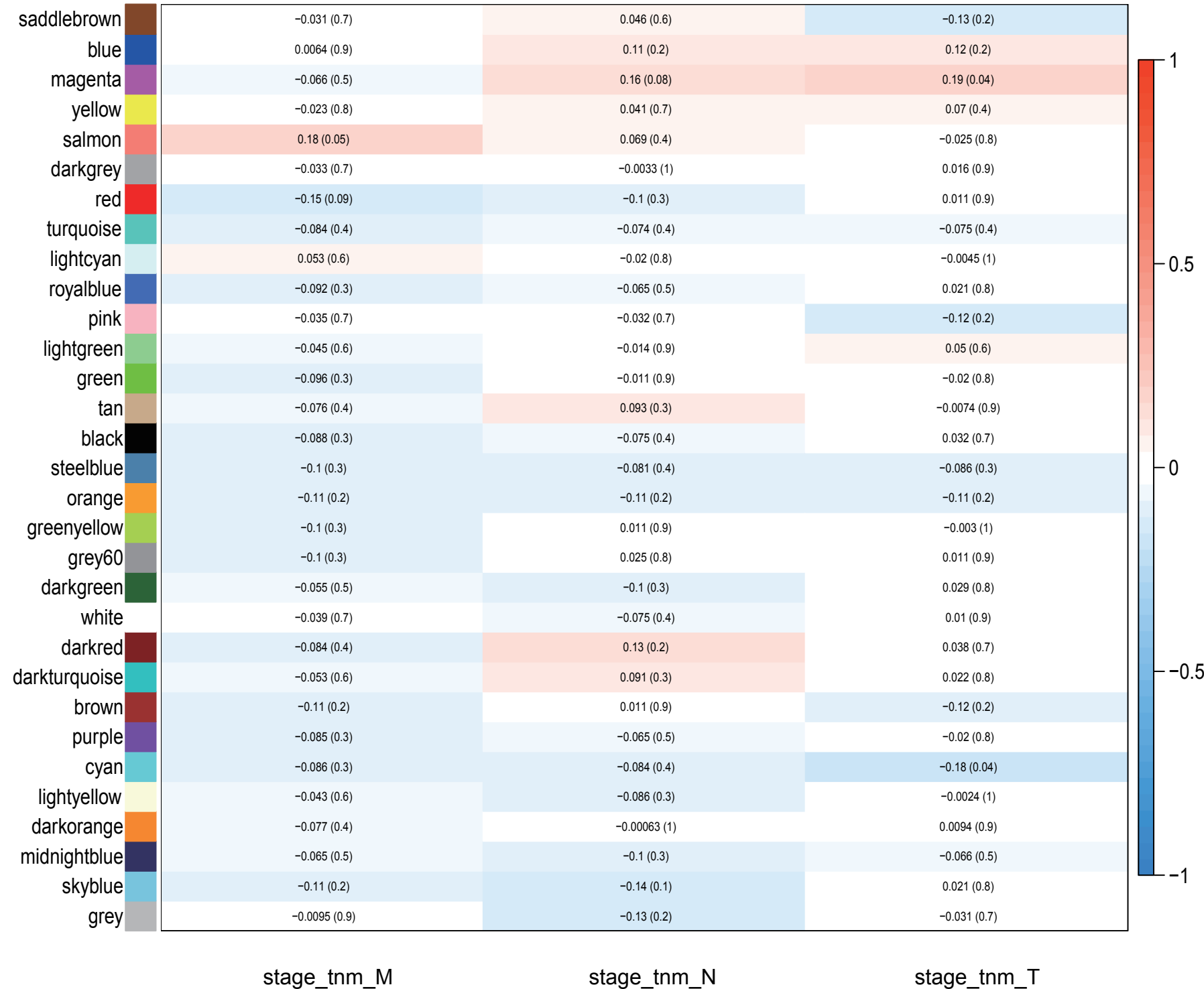

Supplement: Figure S6 [file peerj-07-7696-s006.pdf]

(K) STAD.Module-trait relationships

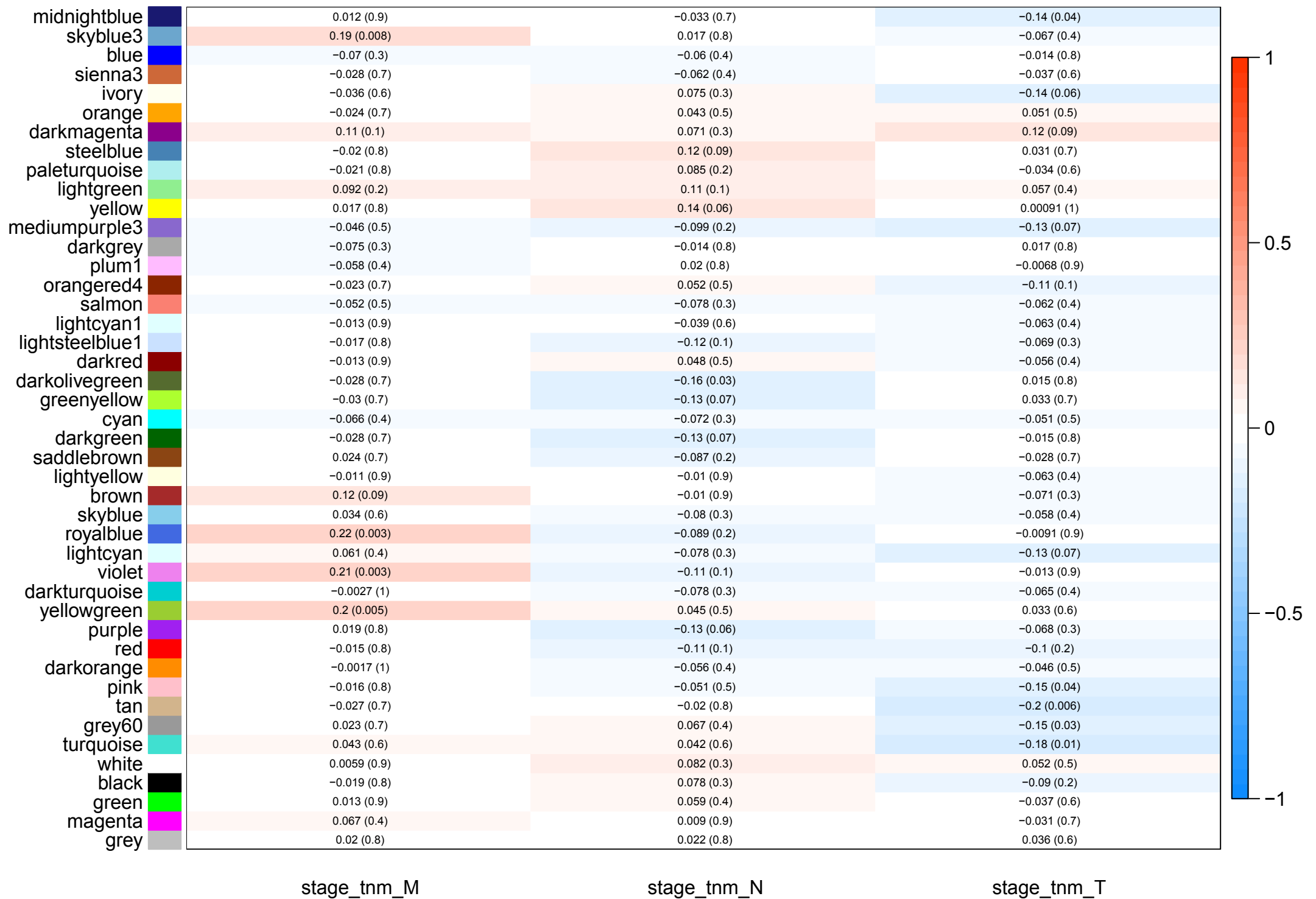

Supplement: Figure S7 [file peerj-07-7696-s007.pdf]
